# Charge Carrier Mobility Improvement in Diketopyrrolopyrrole Block-Copolymers by Shear Coating

**DOI:** 10.3390/polym13091435

**Published:** 2021-04-29

**Authors:** Kristina Ditte, Nataliya Kiriy, Jonathan Perez, Mike Hambsch, Stefan C. B. Mannsfeld, Yulia Krupskaya, Ramesh Maragani, Brigitte Voit, Franziska Lissel

**Affiliations:** 1 Leibniz-Institut für Polymerforschung Dresden e.V., Hohe Straße 6, 01069 Dresden, Germany; ditte@ipfdd.de (K.D.); kiriy-nataliya@ipfdd.de (N.K.); voit@ipfdd.de (B.V.); 2 Faculty of Chemistry and Food Chemistry, Technische Universität Dresden, 01062 Dresden, Germany; 3 Center for Advancing Electronics Dresden, Faculty of Electrical and Computer Engineering, Technische Universität Dresden, Helmholtzstraße 18, 01069 Dresden, Germany; jonathan.perez_andrade1@mailbox.tu-dresden.de (J.P.); mike.hambsch@tu-dresden.de (M.H.); stefan.mannsfeld@tu-dresden.de (S.C.B.M.); 4 Leibniz Institute for Solid State and Materials Research, Helmholtzstraße 20, 01069 Dresden, Germany; y.krupskaya@ifw-dresden.de; 5 Martin-Luther-Universität Halle-Wittenberg, Naturwissenschaftliche Fakultät II, Von-Danckelmann-Platz 4, 06120 Halle, Germany; ramesh.maragani@chemie.uni-halle.de; 6 Institute of Organic Chemistry and Macromolecular Chemistry, Friedrich Schiller University Jena, Humboldtstr 10, 07743 Jena, Germany

**Keywords:** block copolymers, organic field-effect transistors, shear coating, shear speed, thickness-dependent mobility

## Abstract

Shear coating is a promising deposition method for upscaling device fabrication and enabling high throughput, and is furthermore suitable for translating to roll-to-roll processing. Although common polymer semiconductors (PSCs) are solution processible, they are still prone to mechanical failure upon stretching, limiting applications in e.g., electronic skin and health monitoring. Progress made towards mechanically compliant PSCs, e.g., the incorporation of soft segments into the polymer backbone, could not only allow such applications, but also benefit advanced fabrication methods, like roll-to-roll printing on flexible substrates, to produce the targeted devices. Tri-block copolymers (TBCs), consisting of an inner rigid semiconducting poly-diketo-pyrrolopyrrole-thienothiophene (PDPP-TT) block flanked by two soft elastomeric poly(dimethylsiloxane) (PDMS) chains, maintain good charge transport properties, while being mechanically soft and flexible. Potentially aiming at the fabrication of TBC-based wearable electronics by means of cost-efficient and scalable deposition methods (e.g., blade-coating), a tolerance of the electrical performance of the TBCs to the shear speed was investigated. Herein, we demonstrate that such TBCs can be deposited at high shear speeds (film formation up to a speed of 10 mm s^−1^). While such high speeds result in increased film thickness, no degradation of the electrical performance was observed, as was frequently reported for polymer−based OFETs. Instead, high shear speeds even led to a small improvement in the electrical performance: mobility increased from 0.06 cm^2^ V^−1^ s^−1^ at 0.5 mm s^−1^ to 0.16 cm^2^ V^−1^ s^−1^ at 7 mm s^−1^ for the TBC with 24 wt% PDMS, and for the TBC containing 37 wt% PDMS from 0.05 cm^2^ V^−1^ s^−1^ at 0.5 mm s^−1^ to 0.13 cm^2^ V^−1^ s^−1^ at 7 mm s^−1^. Interestingly, the improvement of mobility is not accompanied by any significant changes in morphology.

## 1. Introduction

In recent years, polymer conjugated semiconductors (PSCs), especially donor-acceptor (D-A) copolymers, have been intensely researched for organic electronics applications, e.g., organic field-effect transistors (OFETs) [1]. Because of extensive π-π stacking and overlapping areas of the alternating copolymer, OFETs incorporating D-A PSCs as active materials possess high charge carrier mobilities as compared to PSC homopolymers (e.g., poly(3-hexylthiophene), P3HT), reaching up to 10 cm^2^ V^−1^ s^−1^ [2,3]. In general, PSCs are easily processable from a solution, paving the way for the low-cost fabrication of large-area devices, e.g., via roll-to-roll deposition on solid/flexible substrates [4,5]. The solution processing parameters, such as deposition rate, concentration, choice of solvent, and surrounding temperature, significantly affect the molecular order and charge transport properties of polymer chains. Therefore, along with tuning the chemistry of PSCs [6], the solution deposition parameters have to be optimized to achieve printed high-performance OFETs.

Some of the solution processed deposition techniques include spin coating [7], dip coating [8], shear coating [9,10], and inkjet printing [11]. Because of the simplicity of the setup and flexibility of testing different parameters, as well as the potential to achieve high-performance organic FETs, shear coating is the most ubiquitously used technique within the research community. In shear coating, the active material (organic semiconductor or PSC) is confined between a top movable blade and a temperature-controlled substrate. By moving the blade at a fixed speed, the material is translated across the substrate, leading to a guided and controlled film deposition. When compared to spin coating, solution sheared PSC films have a higher crystallinity and improved molecular packing, which, in turn, facilitates an effective charge transport in OFETs [10,12]. These film properties can be influenced by shear coating parameters, such as shear speed [13], stage temperature and gap between blade [14,15], and substrate. Besides this, there is a wide range of established approaches to control the final film morphology, which can be divided into the ink formulation pre-processing methods [16,17,18], post-processing alignment methods [19,20], substrate pattering [21], as well as modified shear coating geometries methods [22,23].

Post-processing methods were demonstrated to significantly improve the device performance. Some of these methods include the mechanical deformation of a PSC’s film to induce chain alignment in a particular direction [19], or an additional annealing step to improve the thin-film crystallinity and out-of-plane alignment [20]. However, pre-processing techniques are known to be the most effective for controlling a PSC’s alignment. The polymer can be treated with ultraviolet light (UV) [16,17] and/or aged, i.e., the solution processed after a wating period [18], to induce the aggregate/fibril formation, after which the alignment is achieved through blade movement.

Despite achieving high charge carrier mobilities, PSCs are still prone to mechanical failure upon stretching (>30% strain) [24], which limits their electronic functionalities [25]. Several approaches have been followed to overcome this issue, one of which is the utilization of polymer blends [26,27]. Such blends consist of a PSC that is embedded into an elastomeric matrix. The main issue associated with the blending technique is a potentially severe phase segregation of the two components (semiconductor and elastomer) due to their low entropy of mixing, which can affect the electronic performance in the final OFET device [28,29]. Instead of using a bulk blend, it is favorable to form so-called interconnective PSC networks within the elastomeric matrix. The work of Bao et al. showed that the interconnectivity of PSC nanowires at both the top and bottom surfaces of an elastomeric matrix allows for maintaining the charge transport without severe crack formation at 100% strain [30]. Reichmanis et al. utilized less than 1 wt% of PSC to form interpenetrating networks for large-area stretchable FETs arrays, which can maintain their charge mobilities, even after 300 stretching cycles up to 100% strain [26].

Along with the improvement of the film’s mechanical compliance, the blending technique was shown to induce the alignment of PSC chains within the elastomer matrix when shear coating is used for processing. The work of Chang et al. was one of the first to demonstrate a better alignment of a P3HT/polystyrene (PS) system with around 20 wt% of P3HT nanowires as compared to the pure nanowires of P3HT [29]. Here, the final orientation and alignment of polymer chains not only depends on the standard shear parameters, but also on the PSC: elastomer ratio [31], and on the molecular weight of the incorporated PSC [32]. An increase in charge carrier mobilities is observed because the PSCs chains are aligned in the elastomer matrix when shearing. Xu et al. further improved this by forming a multi-scale order alignment when implementing a microtrench blade during the shear process [23].

**Figure 1:**
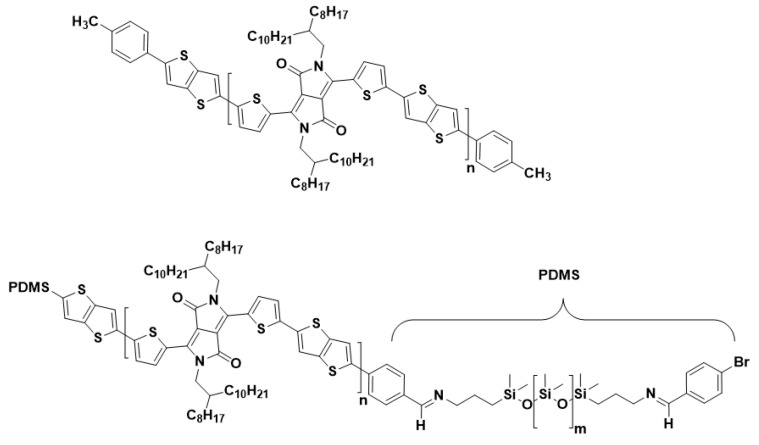
Polymer structure of (top) the reference (0 wt% PDMS), and (bottom) TBCs with 24 and 37 wt% PDMS.

Despite the improved mechanical properties, the crucial and tedious choice of the blend ratios (additional pre-treatment, e.g., UV or aging), as well as the risk of delamination of the PSC from the elastomer matrix, are considered to be the main issues of the blending approach [31,33]. Molecular engineering, which is directed towards developing intrinsically stretchable PSCs, is another route to achieve mechanical compliant PSCs [34,35]. Among the variety of existing methods to tune the electrical/mechanical properties on a molecular level [27], the utilization of block copolymers is conceptually close to the blending approach. Block copolymers consist of two polymers that are connected at their chain ends through a chemical bond, where a phase segregation with distinct properties of each block can be expected due to their thermodynamic incompatibility. Such block copolymers can be built from a conjugated (e.g., semiconducting) and a soft (e.g., elastomeric) segment [36,37,38]. Because the two components are covalently bonded, the issue of delamination of the PSC from the elastomeric matrix is avoided as compared to the physical blending of both moieties. Wang et al. demonstrated the synthesis and characterization of P3HT-block-poly(butyl acrylate) (P3HT-b-PBA) di-block copolymers. The electrical properties of OFETs based on this structure maintained high charge carrier mobilities up to 1000 stretching cycles [37]. In our recent work, triblock copolymers (TBCs) consisting of an inner semiconducting PDPP-TT and two outer soft PDMS blocks were achieved through Stille coupling. While the content of the insulating PDMS block is very high, making up to 67 wt% of the block copolymer structure, the TBC possess relatively high charge carrier mobilities in the same range as the reference PDPP-TT copolymer, and it withstands numerous stretching cycles to 50% strain (up to 1500 cycles) without losing electrical functionality (Figure 1) [36].

While great efforts have been made to understand the behavior of pure PSCs and PSC/elastomer blends upon shear coating, to the best of our knowledge investigations on the influence of shear parameters on the morphology and electrical performance of semiconducting block copolymers is missing as of yet. Yet, understanding these issues is essential in assessing future applications of the TBCs in wearable devices and biosensors, the manufacturing of which should involve cheap and scalable deposition methods, such as doctor blading, ink-jet printing, or spay-coating [7,11,39]. The electrical performance of the devices must tolerate an increased thickness of the active layer in order to use these methods successfully, as the fabrication of ultra-thin, yet highly homogeneous films is still a highly challenging task [22]. On the other hand, in many OFETs, a distinct thickness-dependent performance is observed, and thicker devices frequently show worse characteristics due to increased contact barriers and amounts of traps [40].

Given these facts, here we investigate the influence of shear speed and elastomer content on the thickness of the resulting films and the electrical performance of PDMS-(PDPP-TT)-PDMS TBCs by fabricating bottom gate, top contact OFET devices. The obtained results are then compared to the reference copolymer (i.e., pure PDPP-TT). Morphological analysis (AFM/GIWAXS), as well as polarized UV-Vis spectroscopy, were carried out aiming at a deeper understanding of the observed charge carrier mobility improvement.

## 2. Materials and Methods

### 2.1. Materials

The polymers (0, 24 and 37 wt% PDMS) were synthesized after the previously reported protocols [36].

### 2.2. Field-Effect Transistor Fabrication

OFETs were fabricated on n-type doped Si (100) wafers with 300 nm SiO_2_ thermally grown layer as gate dielectric. The substrates were treated with octadecyltrimethoxysilane (ODTMS) according to literature reports [41]. The polymer solutions were prepared by dissolving the corresponding polymers (27 mg/mL triblock copolymers or 20 mg/mL reference PDPP-TT polymer) in chlorobenzene and stirring at 80 °C overnight. For the shear coating, the substrate temperature (100 °C) was controlled by a thermocouple, and the coating speed was varied using a linear motor from Jenny Science. The blade angle was set to 8° with a gap of 100 μm between the substrate and the edge of the blade. Bottom-gate, top-contact transistors were then finished by the thermal evaporation of 50 nm-thick gold electrodes at a vacuum pressure of 10^−7^ mbar (L = 200 μm, W = 4500 μm). The devices were electrically characterized under ambient conditions using a Keysight B1500 semiconductor analyzer.

### 2.3. Charge Carrier Mobility Calculation

The effective field-effect mobilities were extracted using the algorithm that was proposed by Choi et al. that takes the non-linearity of transfer curves and non-zero threshold voltage into account [42]. The reliability factor *r* was calculated, as follows:

$$r_{sat}=\left(\frac{\sqrt{|I_{SD}{|}^{max}}-\sqrt{|I_{SD}{|}^{0}}\;}{{\left|V_{G}\right|}^{max}}\right)/\left(\frac{WC_{i}}{2L}\mu_{sat}\right)=\left(\frac{\sqrt{|I_{SD}{|}^{max}}-\sqrt{|I_{SD}{|}^{0}}\;}{{\left|V_{G}\right|}^{max}}\right)/{\left(\frac{\partial \sqrt{\left|I_{SD}\right|}}{\partial V_{G}}\right)}^{2}$$

where *µ_sat_* is the calculated mobility, *L*, *W*, and *Ci* the channel length, channel width, and dielectric capacitance of the transistor, $|I_{SD}{|}^{max}\;$the experimental maximum of the source-drain current at maximum gate voltage ${\left|V_{G}\right|}^{max}$, and$\;|I_{SD}{|}^{0}$ is the source-drain current at *V*_g_ = 0.

The effective field-effect mobility can then be calculated as:

$$\mu_{eff}=r\times \mu_{claimed}$$

According to Choi et al., a FET following ideal Schockley equations with mobility, as *µ_eff_* would offer the same electrical performance as the reported non-linear transistors with the claimed mobility *µ_claimed_* [43].

### 2.4. Polarized UV-Vis Spectroscopy

Polarized UV-Vis spectroscopy was carried out with an Agilent Cary 5000 spectrophotometer. The polymer films were sheared on glass slides that were treated with ODTMS. These slides were prepared according to literature reports [41].

### 2.5. Atomic Force Microscopy

The microscope (Bruker, Dimension Icon) was operated in tapping mode using silicon-SPM-sensors with spring constant of ca. 42 N/m and resonance frequency of ca 300 kHz with a tip radius <10 nm. The thickness of the polymer layers was measured using a scratch test technique.

### 2.6. Grazing-Incidence X-ray Diffraction

Measurements were carried out at the XRD1 Beamline at the ELETTRA synchrotron in Trieste, Italy. The incidence angle of the beam was 0.12° and the beam energy was 12.399 keV (*λ* = 1 Å). The Dectris Pilatus 2M area detector was placed at a distance of 400 mm from the sample. All of the measurements were taken at ambient condition. The obtained data were analyzed with WxDiff software (c S.C.B.M).

## 3. Results and Discussion

### 3.1. Electrical Performance

Bottom-gate, top-contact (BGTC) OFETs were fabricated via shear coating to probe the effect of different shear speeds on the overall transistor performance of TBCs with varying elastomer content (0, 24, and 37 wt% PDMS). The dielectric-semiconductor interface was modified with a self-assembled monolayer (SAM) of ODTMS. SAM functionalized surfaces are commonly used for the deposition of organic semiconductors during the fabrication of OFETs due to a substantial increase of charge carrier mobilities by several orders of magnitude. The reason for that is assigned to higher polaron delocalization and better molecular ordering [41,42,44], the dipole-induced built-in electric field of a SAM [45], or the reduction of charge carrier trap states caused by water [46]. However, because of the hydrophobic nature of the used SAMs, the shear speed is limited by wetting conditions and film instabilities (e.g., stick-and-slip instabilities [47]), which might lead to the complete absence of film formation. Not only this, but Hambsch et al. demonstrated a clear negative trend, i.e., that increasing the shear speed leads to decreased charge carrier mobilities of PDPP-based OFETs [42]. The two competing factors, i.e., increase in the charge transport and instabilities of the fluid dynamical meniscus, are among the main obstacles in achieving an increase of the fabrication throughput of semiconducting polymer-based OFET devices [48,49].

**Figure 2:**
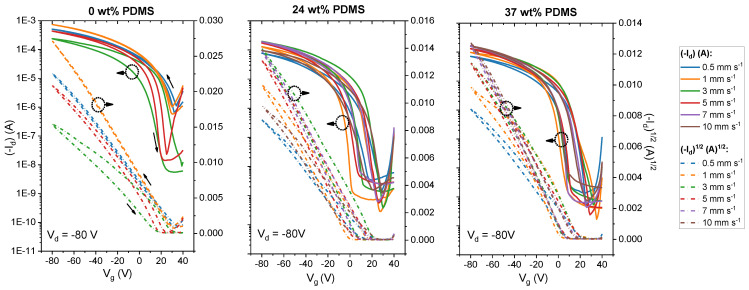
Transfer curves of studied polymers with different wt% of elastomeric PDMS (0, 24 and 37 wt% PDMS) sheared with speeds from 0 to 10 mm s^−^^1^. Solid lines denote the drain current at *V*_d_ = −80 V, while the dashed lines denote the square root of the drain current at *V*_d_ = −80 V.

We first carried out experiments with the reference polymer with 0 wt% PDMS in order to investigate the behavior of the TBCs. Figure 2 (upper panel) represents the dual-sweeping transfer curves of the tested devices. As can be seen in Figure 2, for the reference polymer, the shape of the transfer curves changes significantly when the shear speed is increased. Overall, the threshold voltage (*V*_th_) shifts to more negative voltages in the speed range from 0.5 to 5 mm s^−1^. It is important to note that the reference polymer shows instabilities in the film deposition at 5 mm s^−1^ and that, at higher shear speeds, films are no longer forming. Although the ON/OFF ratio is increased for the transfer curves at higher shear speeds (>2 mm s^−1^), the overall drain current decreased in its value. Additionally, the hysteresis of the curve becomes stronger at 3 mm s^−1^ shear speed. This is in agreement with prior reports [42].

By contrast, both TBCs (24 and 37 wt% PDMS) form stable films up to shear speeds of 10 mm s^−1^. Regarding the shape of the transfer curves, OFETs that are based on either TBC show smaller *V*_th_ values, along with a less pronounced shifting of *V*_th_ upon increasing the shear speed. Similarly, the ON/OFF ratio is maintained within 10^4^ for either TBC throughout the probed shear speed range. This is not only in contrast to the reference polymer without elastomer content, but also to previous work on blends of DPP-based polymers in an elastomer matrix: here, the ON/OFF ratio was reported to change within orders of magnitude while increasing the shear speed [14].

Table 1 summarizes more detailed device characteristics, including effective field-effect mobilities, and *V*_th_ and ON/OFF ratios.

**Figure 3:**
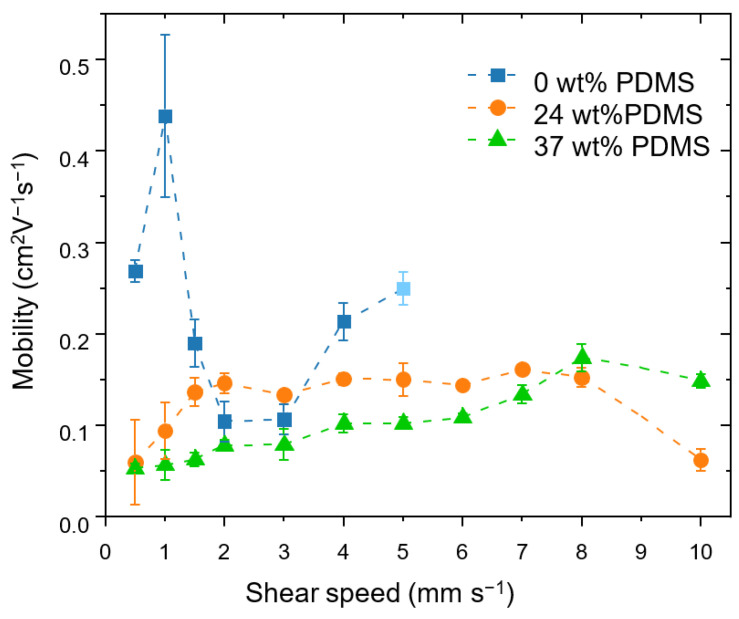
Effective field-effect mobilities achieved at different shear speeds for the studied polymers with different wt% of elastomeric PDMS (0, 24 and 37 wt% PDMS). The error bars represent the standard deviation obtained from four measurements on at least two different substrates at each shear speed. At a shear speed of 5 mm s^−1^, the reference polymer no longer forms films reliably, which is indicated by the light-blue colored value point.

The effective field-effect mobilities (Figure 3) were calculated while taking into account the non-linearity of transfer curves and non-zero threshold voltage, as outlined in the experimental section [43]. More details on dependency of saturated mobility upon *V*_g_ can be found in the Supporting Information (Figures S1–S3 and Table S1 in Supplementary Materials).

The reference polymer shows an insignificant variation of the mobility in the shear rates from 0.5 to 5 mm s^−1^ from 0.27 cm^2^ V^−1^ s^−1^ at 0.5 mm s^−1^ to 0.25 cm^2^ V^−1^ s^−1^ at 5 mm s^−1^, which is consistent with prior reports [42]. At a shear speed of 5 mm s^−1^, the reference polymer no longer forms films reliably and, at higher speeds, the film deposition no longer took place.

A different behavior is observed for the TBCs. Here, increasing the shear speed leads to a moderate increase in carrier mobility: for the TBC with 24 wt% PDMS, the mobility nearly triples, from 0.06 cm^2^ V^−1^ s^−1^ at 0.5 mm s^−1^ to 0.16 cm^2^ V^−1^ s^−1^ at 7 mm s^−1^. The TBC with 37 wt% PDMS similarly displays an increase in mobility, which rises from 0.05 cm^2^ V^−1^ s^−1^ at 0.5 mm s^−1^ to 0.13 cm^2^ V^−1^ s^−1^ at 7 mm s^−1^. At a speed of 8 mm s^−1^, the carrier mobility of the TBC with 37 wt% PDMS reaches the same level as the TBC with 24 wt% PDMS, namely up to 0.17 cm^2^ V^−1^ s^−1^. The same trend is seen for the maximum drain current at *V*g = −80 V (see Supporting Information, Figure S4). At an even higher speed of 10 mm s^−1^, the mobility of the TBC with 37 wt% PDMS stays within the same order of magnitude, while the TBC with 24 wt% PDMS exhibits a sudden drop in mobility due to the formation of holes in the film (see Supporting Information, Figure S5). The small charge mobilities at low shear speeds for TBCs might be attributed to the devices having a high surface roughness and inhomogeneous coverage on the substrate (see Supporting Information, Table S2).

It is important to emphasize that the increase of the speed of the shear coating from 1 to 10 mm s^−1^ is accompanied by a steady increase of the film thickness (from approximately 50 nm for 24 wt% PDMS and 150 nm for 37 wt% PDMS up to 400 nm for both TBCs). Therefore, the increased mobility could be ascribed to the thickness: Xu and co-workers observed a similar behavior using blends of semiconducting and elastomer polymers (however, the thickness values are lower than in the present work) [23]. The tolerance of the TBCs’ mobility to increased film thickness is an attractive feature for applications targeting high-throughput deposition methods (e.g., doctor blading), as, here, achieving high quality films with precisely controlled low thicknesses is highly challenging.

Additional spectral and morphological studies were carried out aiming at a better understanding of the observed dependence of the TBCs’ electrical performance on the deposition conditions. As reported in the literature, the most common reasons for changes of the electrical performance are morphological variations at different scales: (i) large-scale chain-orientation order [50]; (ii) macroscopic features, e.g., presence or absence of major inhomogeneities, pin-holes, cracks, variation of roughness, thickness [40]; and, (iii) variations of the micro-structure, e.g., molecular packing and crystallinity [21]. Here, we used polarized UV-Vis-NIR spectroscopy, atomic force microscopy, as well as grazing-incidence X-ray diffraction to gain deeper insight into morphological changes.

### 3.2. Polarized UV-Vis-NIR

The increased mobilities could be connected to the formation of better oriented and, hence, more anisotropic films. To correlate the electrical performance with the chain-orientation, polarized UV-Vis-NIR studies were carried out to analyze optical absorption anisotropies in parallel and perpendicular directions of the polarized beam towards the shear direction. With this technique, it is possible to probe the preferences in polymer chain alignment. In general, an equal absorption in both directions demonstrates a random average orientation of the chains, while the higher absorption in either a parallel or perpendicular direction indicates a higher alignment in this particular direction.

**Figure 4:**
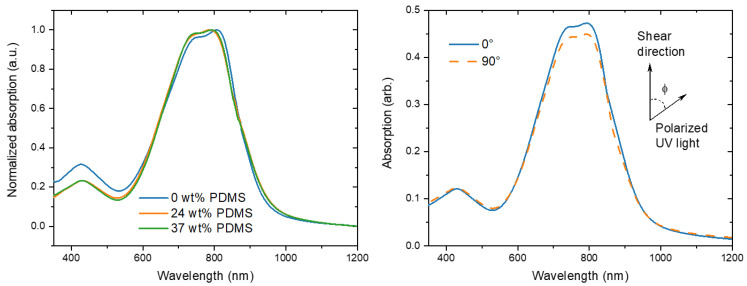
(**Left**) normalized UV-Vis spectra of the studied polymers with different wt% of elastomeric PDMS (0, 24, and 37 wt% PDMS). (**Right**) typical polarized UV-Vis spectra of TBC with 37 wt% PDMS prepared at 1 mm s^−1^ shear speed.

For the reference PDPP-TT polymer, three main peaks are observed at around 430, 747, and 807 nm (Figure 4). Because the conjugated backbone of the TBCs is equivalent to the reference polymer, similarly the main peaks were observed at 430, 745, and 789 nm. The onset of absorption of the TBCs is slightly red-shifted as compared to the reference polymer, which is reminiscent of PDPP-TT/elastomer blends [14].

**Figure 5:**
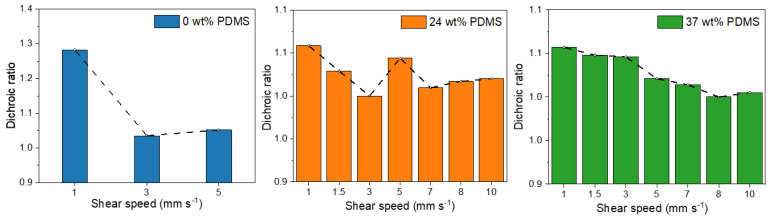
Dichroic ratios of the studied polymers with different wt% of elastomeric PDMS (0, 24, and 37 wt% PDMS) sheared at different speeds.

The dichroic ratio was calculated (A_0°_/A_90°_) for the polarized absorption spectra to evaluate the polymer chain alignment in each polymer film at different shear speeds (Figure 5).

For the fully conjugated reference copolymer, the dichroic ratio is around 1.3 for 1 mm s^−1^, which is consistent with literature reports of PDPP-TT [14], but lower than that observed for DPP polymers with less rigid donor units [10]. When the shear speed is increased, the dichroic ratio is decreasing to 1.0 for 3 mm s^−1^, indicating a decline of chain orientation, consistent with the observed decline of charge carrier mobility. In contrast, while the initial dichroic ratio of the TBCs is slightly lower, in the range of 1.1–1.0, the dichroic ratio is nearly maintained when the shear speed is increased up to 10 mm s^−1^. Only a tentative negative trend is observed for the TBC with 37 wt% PDMS, here the dichroic ratio declines from 1.1 for 1 mm s^−1^ to 1.0 for 10 mm s^−1^. Hence, the observed increase in charge carrier mobility for higher shear speeds (Table 1 and Figure 5) is not caused by an increase in the chain alignment.

### 3.3. Thickness Measurements

In the work of Le Berre et al., two regimes of shear deposition were identified: the evaporative and Landau-Levich regime [51]. For the former, the film formation happens directly at the contact line with the blade. In this regime, higher shear speeds lead to a thinner film being deposited on the substrate. In the Landau–Levich regime, the wet polymer film is stretched by viscous forces before subsequent drying, leaving behind relatively thick films. The shear speed at which the transition between the two regimes occurs depends on a variety of factors, e.g., solution concentration, deposition temperature, choice of solvent. In general, the thicker films deposited in the Landau-Levich regime exhibit a lower mobility, yet for industrial scale up, high coating speeds (e.g., >1 m min^−1^) might be desirable [52].

**Figure 6:**
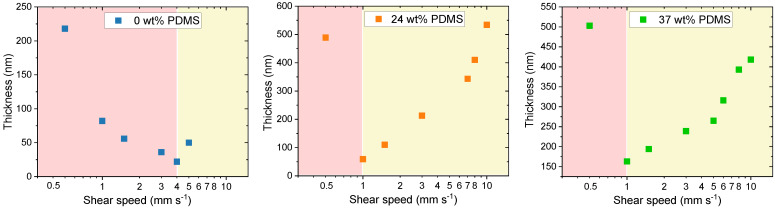
Thickness of the studied polymers with different wt% of elastomeric PDMS (0, 24 and 37 wt% PDMS). The light-red block defines the evaporative deposition regime, while the light-yellow block denotes the Landau-Levich deposition regime.

As expected, two deposition regimes were observed for all of the analyzed polymers. For the reference polymer (0 wt% PDMS), the critical point is at a shear speed of 4 mm s^−1^, where the transition between both regimes is happening, consistent with previous reports [12]. Close to this speed, the deposited films were quite thin, around 50 nm (Figure 6). However, for the TBCs with 24 or 37 wt% PDMS, the transition between the regimes was observed at a slower shear speed of around 1 mm s^−1^. The same behavior was already described for blends of semiconducting DPP-based polymer with an elastomer matrix [23].

It has to be noted that, in the Landau–Levich regime, the films not only thicken, but that an improvement in polymer chain alignment is not expected as the polymer aggregates or its free chains relax before their orientation can be fixed in the final film morphology [12]. Despite this, we observe an increase in the charge carrier mobilities for the TBCs with 24 or 37 wt% PDMS within the Landau–Levich deposition regime. The ability of the TBCs to maintain charge carrier mobilities upon shearing at high shear speeds might be beneficial for organic photovoltaics (OPVs), as, here, a large active layer thickness toleration is required, especially when targeting commercial production [53]. While the variation of thickness could thus be a reason for the observed relative mobility increase, the exact mechanistic reason behind this dependence is not understood as of yet.

### 3.4. Atomic Force Microscopy

**Figure 7:**
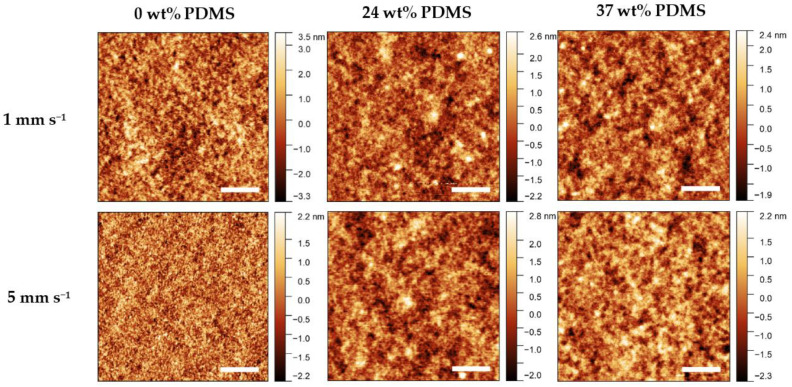
AFM height images of the studied polymers with different wt% of elastomeric 0 wt% PDMS, 24 wt% PDMS, and 37 wt% PDMS sheared at 1 mm s^−1^ (**upper panel**) and 5 mm s^−1^ (**lower panel**). The scale bar denotes 1 µm.

Atomic Force Microscopy (AFM) was performed to observe changes in morphology caused by different shear speeds (Figure 7). A general observation is that all of the investigated films prepared at different shear speeds are rather smooth and contain no visual defects or major inhomogeneities.

For the reference polymer, the root-mean-square roughness (RMS) decreases rapidly from 1.2 nm to approximately 0.7 nm when increasing the shear speed from 1 to 5 mm s^−1^. The fiber-like structures (grain size) decrease in size from 1 to 5 mm s^−1^, which is consistent with the decrease in effective field-effect mobility at high shear speeds. By contrast, when the shear speed is increased from 1 to 5 mm s^−1^, the RMS value remains relatively constant, at 0.6–0.7 nm, for either TBC. Further information on the RMS values and AFM images can be found in the Supporting Information (Table S2). As such, the variation of morphology, as assessable by AFM, cannot be a reason for the different electrical performance of the TBCs.

### 3.5. Grazing-Incidence X-ray Diffraction

**Figure 8:**
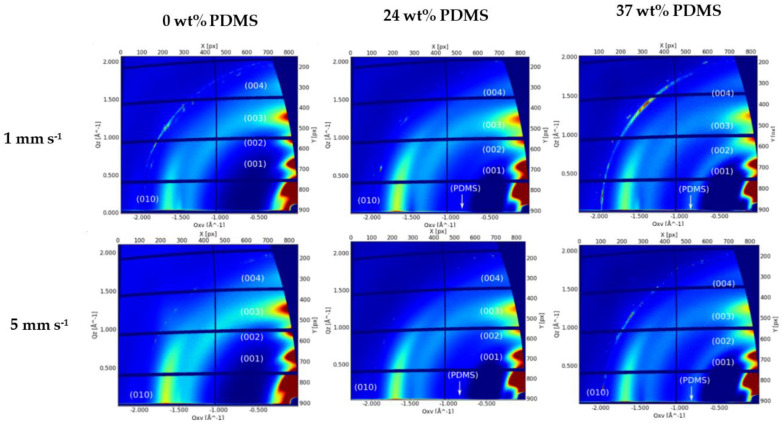
GIWAXS patterns of the studied polymers with different wt% of elastomeric PDMS: AFM height images of the studied polymers with different wt% of elastomeric 0 wt% PDMS, 24 wt% PDMS, and 37 wt% PDMS sheared at 1 mm s^−1^ (**upper panel**) and 5 mm s^−1^ (**lower panel**).

Molecular packing, crystallinity, and chain orientation of all polymer films were investigated using grazing-incidence wide-angle X-ray scattering (GIWAXS) (Figure 8).

The reference polymer (0 wt% PDMS) exhibits a series of (h00) reflections oriented along Q_z_ corresponding to a semicrystalline microstructure with edge-on orientated lamellae. The (100) peak at Q_z_~0.34 Å^−1^ is attributed to a lamellar stacking distance of ~18.48 Å. The (010) peak is observed at Q_xy_ 1.67 Å^−1^, corresponding to a π-π stacking distance of 3.76 Å. A broad amorphous halo is also observed at Q_xy_ 1.4 Å^−1^, thought to be caused by the distance between the Sulphur centers of two adjacent thiophene rings (Figure 8). The GIWAXS patterns of both TBCs’ films exhibit the same features as the pristine polymer with a slight decrease on the lamellar stacking and a slight increase on the π-π stacking distance (3.72 and 19.04 Å, respectively). Additionally, an amorphous halo corresponding to the amorphous PDMS blocks is clearly visible at Q_xy_ 0.84 Å^−1^ (Figure 8). From the analyzed peak positions and calculated coherence length (see Supplementary Information, Table S3), there is no clear difference on the film morphology with the addition of PDMS content or by the increase of the shear speed.

## 4. Conclusions

In summary, we investigated the dependency of the electrical properties of OFETs using TBCs (i.e., PDPP-TT reference polymer end-capped with PDMS moieties) on the shear speed and morphological changes during this process. While the reference polymer with 0 wt% PDMS no longer forms films above a shear speed of 5 mm s^−1^, the TBCs with 24 and 37 wt% PDMS still give stable and electrically functional films at 10 mm s^−1^. When investigating the film thickness, all of the polymers show an evaporative and a Landau-Levich deposition regime, the latter marked by a pronounced thickening of the deposited films. The reference polymer shows the expected decrease in effective field-effect mobility upon increasing the shear speed and obtaining thicker films. In contrast, for both TBCs, a moderate increase in mobility is observed when the shear speed is increased. This mobility increase is taking place, despite the expected film thickening at high shear speeds. Except for the thickness increase, no particular changes were observed in the film morphology (as demonstrated by polarized UV-Vis, AFM, and GIWAXS measurements), indicating that the mobility increase of the TBCs cannot be attributed to a better ordering. The variation of film thickness might be a reason for the relative charge carrier mobility increase; however, the mechanistic reasons behind this phenomenon are not clear as of yet. Overall, the obtained results confirm that the TBCs can be deposited at high speeds without distorting their morphological characteristics, which is reflected in the ability to retain their electric properties. The findings can be potentially applied to allow large throughput deposition methods, for which it is highly challenging to reliably achieve films of low thickness and high quality.

## Figures and Tables

**Table 1:** Parameters of polymers with different wt% of elastomeric PDMS (0, 24 and 37 wt% PDMS) sheared at different speeds. The data are obtained from four measurements on at least two different substrates at each shear speed.

Shear Speed, mm s^−1^	0 wt% PDMS			24 wt% PDMS			37 wt% PDMS		
	*μ*_eff_, cm^2^ V^−1^ s^−1^	*V*_th_, V	*I*_ON/OFF_, A	*μ*_eff_, cm^2^ V^−1^ s^−1^	*V*_t__h_, V	*I*_ON/OF_, A	*μ*_eff_, cm^2^ V^−1^ s^−1^	*V*_th_, V	*I*_ON/OF_, A
0.5	0.27 ± 0.01	31 ± 4	3 × 10^2^ ± 1 × 10^2^	0.06 ± 0.04	17 ± 3	8 × 10^3^ ± 7 × 10^3^	0.05 ± 0.01	21 ± 4	6 × 10^4^ ± 5 × 10^4^
1	0.44 ± 0.09	31 ± 11	1 × 10^3^ ± 2 × 10^2^	0.09 ± 0.03	18 ± 4	5 × 10^4^ ± 2 × 10^4^	0.06 ± 0.02	17 ± 1	4 × 10^4^ ± 3 × 10^4^
1.5	0.19 ± 0.03	30 ± 5	8 × 10^4^ ± 4 × 10^4^	0.14 ± 0.02	24 ± 4	6 × 10^4^ ± 2 × 10^4^	0.06 ± 0.01	22 ± 2	4 × 10^4^ ± 2 × 10^4^
2	0.10 ± 0.02	20 ± 2	5 × 10^4^ ± 2 × 10^4^	0.15 ± 0.01	18 ± 4	3 × 10^4^ ± 2 × 10^4^	0.08 ± 0.01	22 ± 1	4 × 10^4^ ± 2 × 10^4^
3	0.11 ± 0.02	28 ± 2	4 × 10^4^ ± 3 × 10^4^	0.13 ± 0.01	22 ± 4	8 × 10^4^ ± 2 × 10^4^	0.08 ± 0.02	21 ± 2	3 × 10^4^ ± 1 × 10^4^
4	0.21 ± 0.02	27 ± 3	5 × 10^4^ ± 3 × 10^4^	0.15 ± 0.01	24 ± 4	7 × 10^4^ ± 5 × 10^4^	0.10 ± 0.01	18 ± 4	3 × 10^4^ ± 2 × 10^4^
5	0.25 ± 0.02	25 ± 6	3 × 10^4^ ± 9 × 10^3^	0.15 ± 0.02	18 ± 3	6 × 10^4^ ± 5 × 10^4^	0.10 ± 0.01	17 ± 1	2 × 10^5^ ± 7 × 10^4^
6	-	-	-	0.14 ± 0.01	18 ± 3	2 × 10^4^ ± 2 × 10^4^	0.11 ± 0.01	17 ± 1	2 × 10^4^ ± 6 × 10^4^
7	-	-	-	0.16 ± 0.01	18 ± 3	4 × 10^4^ ± 1 × 10^4^	0.13 ± 0.01	17 ± 3	8 × 10^4^ ± 6 × 10^4^
8	-	-	-	0.15 ± 0.01	19 ± 4	9 × 10^4^ ± 9 × 10^4^	0.17 ± 0.02	20 ± 1	5 × 10^4^ ± 2 × 10^4^
10	-	-	-	0.06 ± 0.01	15 ± 5	7 × 10^3^ ± 5 × 10^3^	0.15 ± 0.01	14 ± 1	3 × 10^4^ ± 2 × 10^4^

## Data Availability

The data presented in this study is available on request from the corresponding author.

## Supplementary Materials

The following are available online at https://www.mdpi.com/article/10.3390/polym13091435/s1: Figures S1–S3: gate-voltage dependent saturation mobility and non-linear square root of the transfer characteristic for the reference (0% wt% PDMS), 24 and 37 wt% PDMS content TBCs, respectively. Figure S4: The relationship of drain current at *V*_g_ = −80 V to the shear speed. Figure S5: AFM height image of a film of the TBC with 24 wt% PDMS deposited at 10 mm s^−1^ shear speed. Table S1: summarized μ_sat,max_ and μ_sat,min_ for the studied polymers with different wt% of elastomeric PDMS. Table S2: The roughness of films of the studied polymers with different wt% of elastomeric PDMS. Table S3: GIWAXS spacing parameters for thin films of the studied polymers with different wt% of elastomeric PDMS.
