# Supplementary material for: Charge Carrier Mobility Improvement in Diketopyrrolopyrrole Block-Copolymers by Shear Coating
Source: Polymers (Basel). 2021 Apr 29;13(9):1435. doi: 10.3390/polym13091435 (PMC8125458; doi:10.3390/polym13091435)
Supplement: Supplementary file 1 [file polymers-13-01435-s001.zip › polymers-1184934-supplementary.pdf]

polymers

MDPI

Type of the Paper (Article, Review, Communication, etc.)

Supplementary Materials

Charge carrier mobility improvement in diketopyrrolopyrrole block-copolymers by shear coating

Kristina Ditte[1,2], Nataliya Kiriy[1], Jonathan Perez[3, 4], Mike Hambsch[3], Stefan C. B. Mannsfeld[3], Yulia Krupskaya[4], Ramesh Maragani[5], Brigitte Voit[1,2], and Franziska Lissel[1,2,6*]

[1] Leibniz-Institut für Polymerforschung Dresden e.V., Hohe Straße 6, Dresden 01069, Germany; ditte@ipfdd.de (K.D.), kiriy-nataliya@ipfdd.de (N.K.)
[2] Faculty of Chemistry and Food Chemistry, Technische Universität Dresden, Dresden 01062, Germany
[3] Center for Advancing Electronics Dresden and Faculty of Electrical and Computer Engineering, Technische Universität Dresden, Helmholtzstraße 18, Dresden 01069, Germany; mike.hambsch@tu-dresden.de (M.H.), stefan.mannsfeld@tu-dresden.de (S.C.B.M.)
[4] Leibniz Institute for Solid State and Materials Research, Helmholtzstraße 20, Dresden 01069, Germany; jonathan.perez_andrade1@mailbox.tu-dresden.de (J.P.), y.krupskaya@ifw-dresden.de (Y.K.)
[5] Martin-Luther-Universität Halle-Wittenberg, Naturwissenschaftliche Fakultät II, Von-Danckelmann-Platz 4, 06120 Halle (Saale), Germany; ramesh.maragani@chemie.uni-halle.de (R.M.)
[6] Institute of Organic Chemistry and Macromolecular Chemistry, Friedrich Schiller University Jena, Humboldtstr. 10, 07743 Jena, Germany
* Correspondence: lissel@ipfdd.de (F.L.)

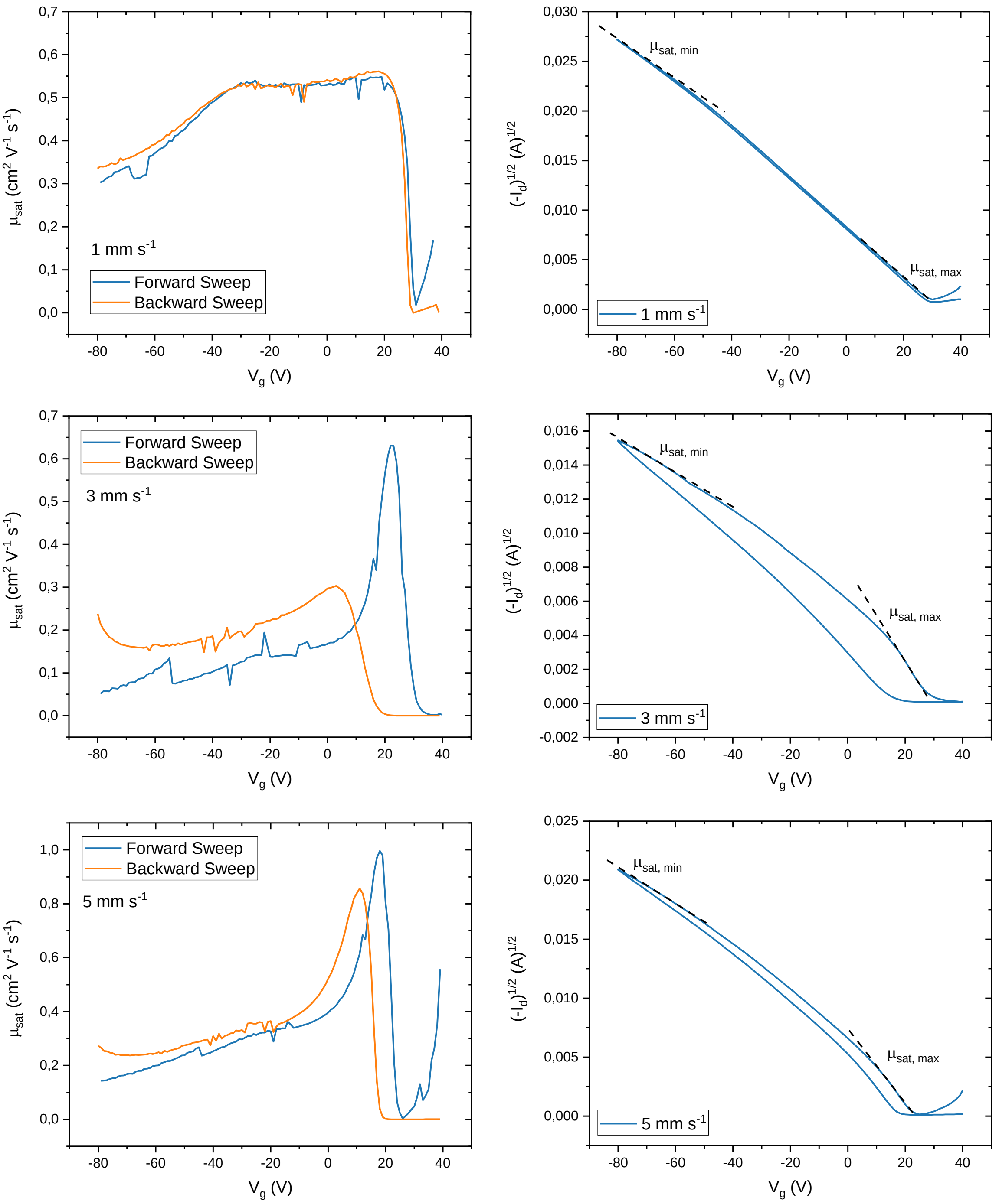

Figure S1. Characteristics of the reference polymer (0 wt% PDMS content) at different shear speeds (from top to bottom: 1 / 3 / 5 mm s^{-1}). *Left side*: gate-voltage dependent saturation mobility. *Right side*: non-linear square root of the transfer characteristic (V_{sd} = -80 V). The black dashed lines indicate the different gate voltage dependent mobility regimes; $\mu_{sat, max}$ is the mobility at gate voltage close to threshold voltage and $\mu_{sat, min}$ is the mobility at high gate voltage (-60 V to -80 V).

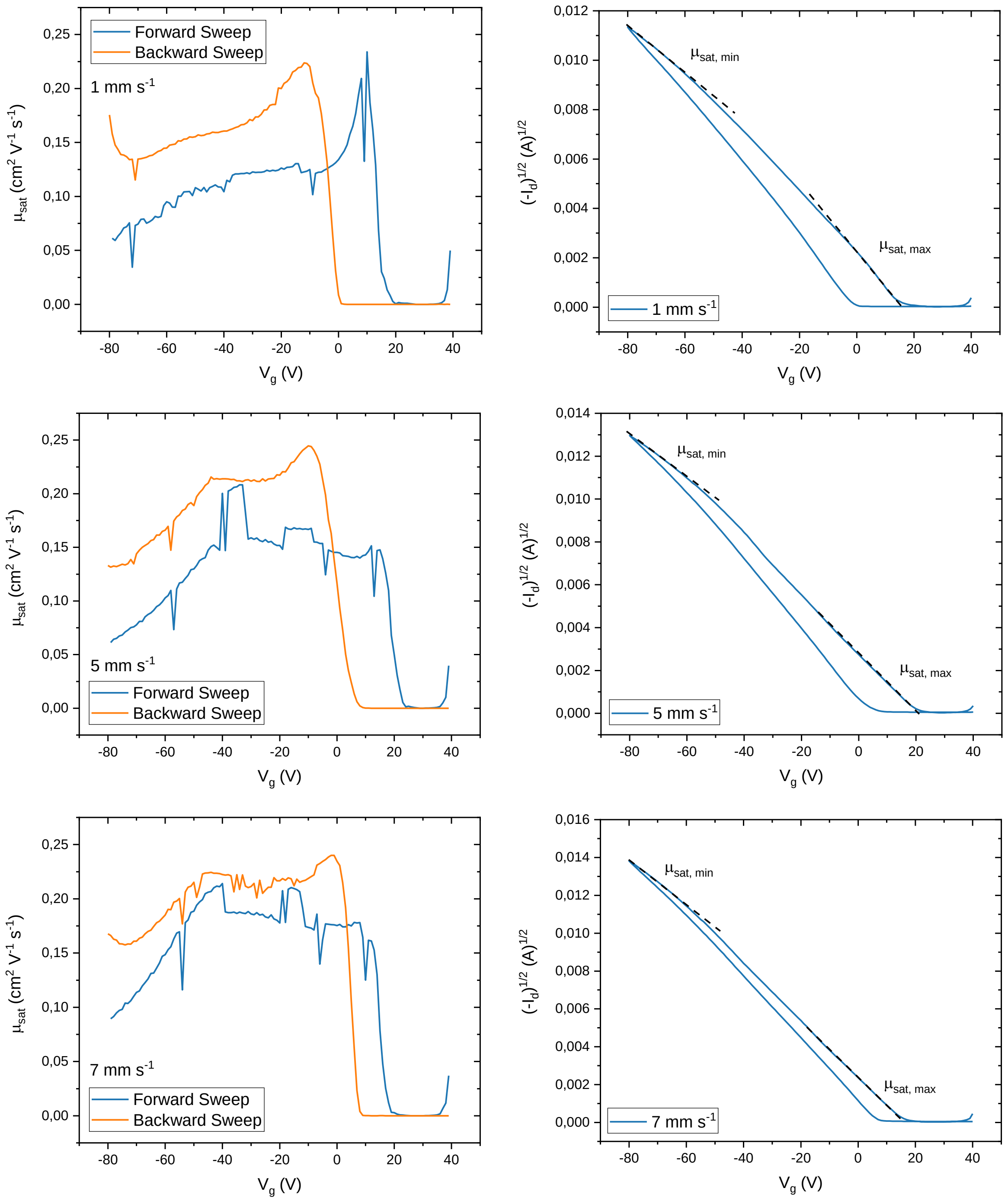

Figure S2. Characteristics of the TBC with 24 wt% PDMS content at different shear speeds (from top to bottom: 1 / 5 / 7 mm s^{-1}). *Left side*: gate-voltage dependent saturation mobility. *Right side*: non-linear square root of the transfer characteristic (V_{sd} = -80 V). The black dashed lines indicate the different gate voltage dependent mobility regimes; $\mu_{sat,\, max}$ is the mobility at gate voltage close to threshold voltage and $\mu_{sat,\, min}$ is the mobility at high gate voltage (-60 V to -80 V).

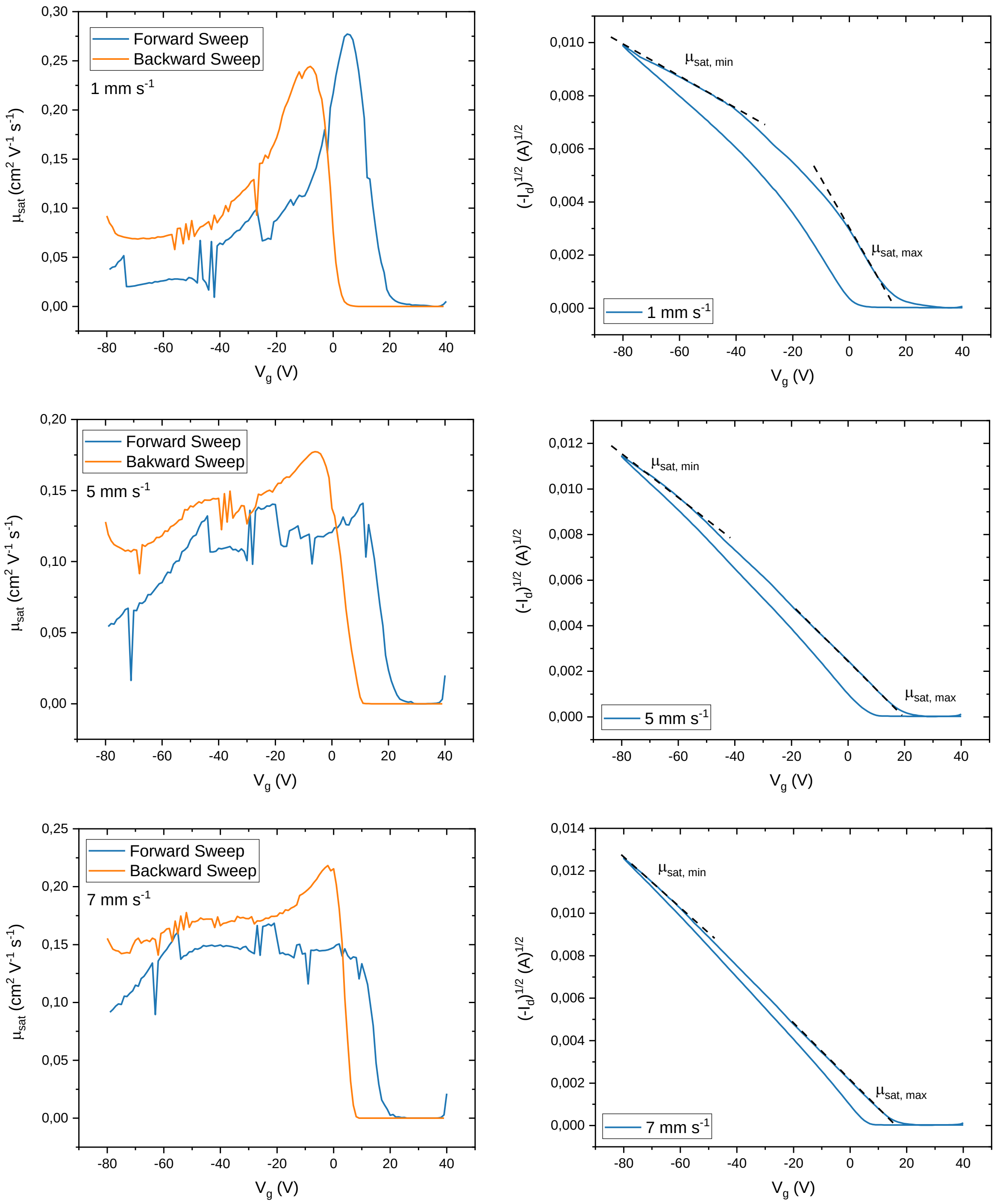

Figure S3. Characteristics of the TBC with 37 wt% PDMS content at different shear speeds (from top to bottom: 1 / 5 / 7 mm s^{-1}). *Left side*: gate-voltage dependent saturation mobility. *Right side*: non-linear square root of the transfer characteristic (V_{sd} = -80 V). The black dashed lines indicate the different gate voltage dependent mobility regimes; $\mu_{sat,\ max}$ is the mobility at gate voltage close to threshold voltage and $\mu_{sat,\ min}$ is the mobility at high gate voltage (-60 V to -80 V).

Table S1. Summarized $\mu_{sat,max}$ and $\mu_{sat,min}$ for the studied polymers with different wt% of elastomeric PDMS (0, 24 and 37 wt% PDMS) sheared from 0.5 to 10 mms^{-1}. The data is obtained from 4 measurements on at least 2 different substrates at each shear speed.

Shear speed, mm s^{-1}	0 wt% PDMS		24 wt% PDMS		37 wt% PDMS	
	$\mu_{sat,max}$, cm^2 V^{-1} s^{-1}	$\mu_{sat,min}$, cm^2 V^{-1} s^{-1}	$\mu_{sat,max}$, cm^2 V^{-1} s^{-1}	$\mu_{sat,min}$, cm^2 V^{-1} s^{-1}	$\mu_{sat,max}$, cm^2 V^{-1} s^{-1}	$\mu_{sat,min}$, cm^2 V^{-1} s^{-1}
0.5	0.31±0.01	0.18±0.01	0.04±0.02	0.03±0.01	0.07±0.02	0.03±0.01
1	0.58±0.17	0.31±0.05	0.12±0.05	0.07±0.02	0.13±0.03	0.04±0.01
1.5	0.31±0.07	0.10±0.02	0.17±0.01	0.10±0.02	0.08±0.01	0.05±0.01
2	0.41±0.06	0.05±0.01	0.18±0.01	0.11±0.01	0.08±0.01	0.06±0.01
3	0.29±0.13		0.16±0.01	0.10±0.01	0.14±0.02	0.06±0.01
4	0.42±0.10	0.11±0.01	0.19±0.02	0.10±0.01	0.15±0.01	0.06±0.01
5	0.40±0.10	0.16±0.02	0.16±0.01	0.11±0.03	0.12±0.01	0.07±0.01
6	-	-	0.14±0.01	0.10±0.01	0.12±0.01	0.08±0.01
7	-	-	0.19±0.01	0.11±0.01	0.15±0.02	0.11±0.01
8	-	-	0.16±0.01	0.11±0.01	0.20±0.01	0.10±0.01
10	-	-	0.06±0.01	0.03±0.01	0.16±0.01	0.11±0.01

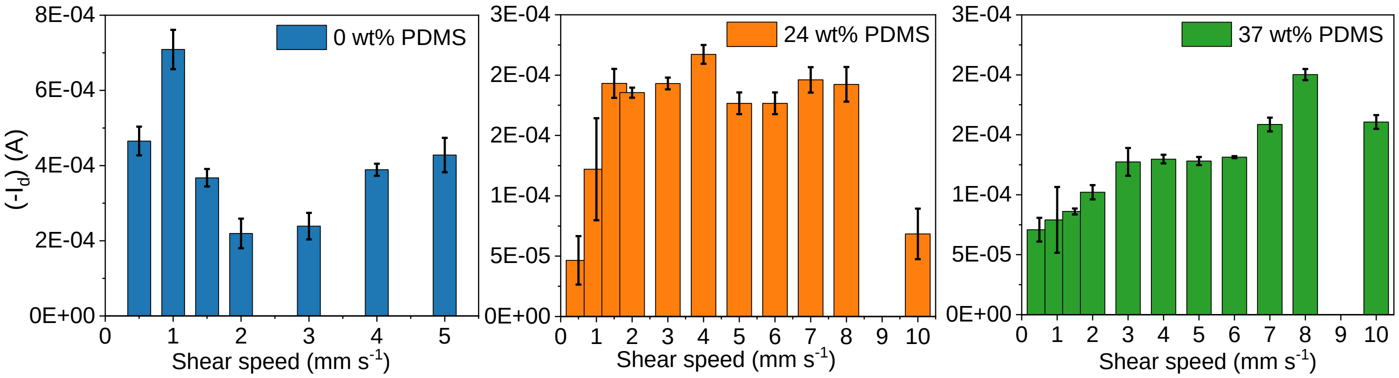

Figure S4. The relationship of drain current at V_g = -80V to the shear speed. The data is obtained from 4 measurements on at least 2 different substrates at each shear speed.

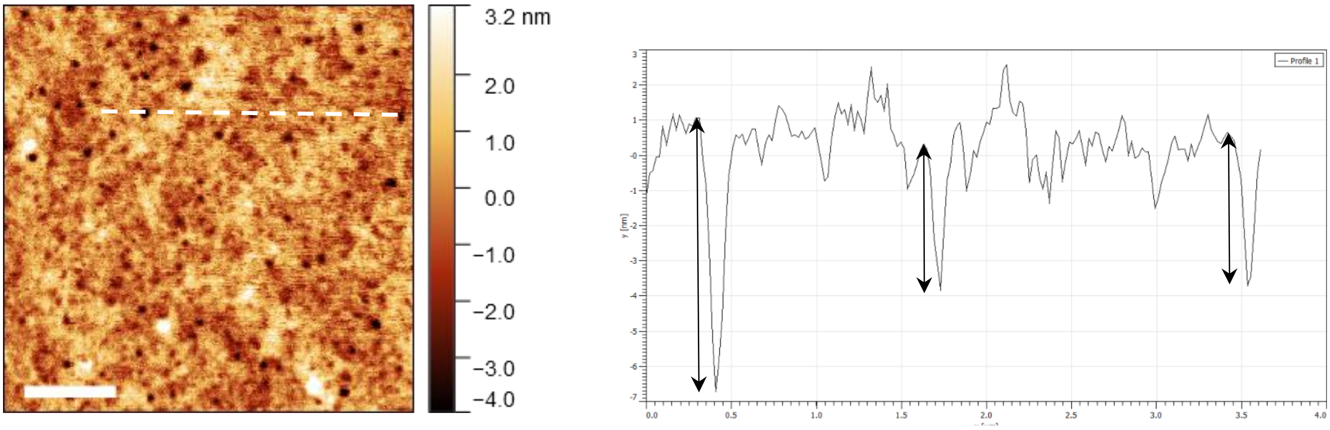

Figure S5. (left) AFM height image of a film of the TBC with 24 wt% PDMS deposited at 10 mm s^{-1} shear speed, the scale bar denotes 1 um; (right) cross-section of the same image, the arrows indicate the presence of holes in the film after the deposition.

Table S2. The roughness of films of the studied polymers with different wt% of elastomeric PDMS (0, 24 and 37 wt% PDMS) sheared at different speeds.

Shear speed, mm s^{-1}	RMS (R_q), nm		
	**0 wt% PDMS**	**24 wt% PDMS**	**37 wt% PDMS**
1	1.10	0.75	0.67
1.5	0.85	0.74	0.67
3	0.78	0.75	0.65
5	0.74	0.72	0.63
7	-	0.70	0.64
10	-	1.00 (due to presence of holes)	0.65

Table S3. GIWAXS spacing parameters for thin films of the studied polymers with different wt% of elastomeric PDMS (0, 24 and 37 wt% PDMS) sheared at different speeds.

			π-π Stacking			Lamellar Packing		
		**Shearing direction**	**Qxy (Å^{-1})**	**d(Å)**	**Coherence length**	**Qz (Å^{-1})**	**d(Å)**	**Coherence length**
0% PDMS	1 mm/s	Parallel	1.68	3.75	1.57	0.34	18.63	105.40
		Perpendicular	1.68	3.74	1.58	0.34	18.73	121.20
	5 mm/s	Parallel	1.67	3.76	1.57	0.34	18.59	121.20
		Perpendicular	1.67	3.76	1.57	0.34	18.72	121.70
24% PDMS	1 mm/s	Parallel	1.69	3.72	1.59	0.33	19.06	102.30
		Perpendicular	1.69	3.71	1.59	0.33	19.04	92.23
	5 mm/s	Parallel	1.69	3.72	1.59	0.33	18.93	84.68
		Perpendicular	1.69	3.71	1.59	0.33	18.93	88.23
	7 mm/s	Parallel	1.69	3.71	1.59	0.33	18.97	94.14
		Perpendicular	1.69	3.71	1.59	0.33	19.04	102.47
37% PDMS	1 mm/s	Parallel	1.69	3.72	1.59	0.33	19.07	101.59
		Perpendicular	1.69	3.72	1.59	0.33	19.01	86.42
	5 mm/s	Parallel	1.69	3.71	1.59	0.33	19.01	100.72
		Perpendicular	1.68	3.73	1.58	0.33	19.05	100.04
	7 mm/s	Parallel	1.69	3.72	1.59	0.33	19.03	89.43
		Perpendicular	1.69	3.71	1.59	0.33	18.91	71.11
